# Supplementary material for: Tomato Spotted Wilt Virus Suppresses the Antiviral Response of the Insect Vector, Frankliniella occidentalis, by Elevating an Immunosuppressive C18 Oxylipin Level Using Its Virulent Factor, NSs
Source: Cells. 2024 Aug 19;13(16):1377. doi: 10.3390/cells13161377 (PMC11352781; doi:10.3390/cells13161377)
Supplement: Supplementary file 1 [file cells-13-01377-s001.zip › cells-3155240-supplementary.pdf]

Supplementary Information

Table S1. GenBank accession numbers and abbreviations used for phylogenetic analysis of EpOME synthase *CYP* genes.

Table S2. GenBank accession numbers and abbreviations used for phylogenetic analysis of *sEH*

Table S3. Sequences of primers used in this study and their PCR conditions

Table S4. Probe sequences used in FISH assay

Figure S1. LC-MS/MS of EpOMEs and DiHOMEs of *F. occidentalis*

Figure S2. FISH assay of TSWV titer in *F. occidentalis* in response to arachidonic acid (AA) and AUDA using sense probe specific to TSWV N. the contours are drawn because of no signal. The scale bars represent 0.1mm.

Table S1. GenBank accession numbers and abbreviations used for phylogenetic analysis of EpOME synthase CYP genes.

Species	Abbreviation	Accession number
Frankliniella occidentalis	Fo CYP1	AED99066
Frankliniella occidentalis	Fo CYP2	AED99065
Frankliniella occidentalis	Fo CYP3	KAE8749876
Frankliniella occidentalis	Fo CYP4	KAE8739041
Frankliniella occidentalis	Fo CYP5	XP052132944
Frankliniella occidentalis	Fo CYP6	XP052131731
Frankliniella occidentalis	Fo CYP8	XP026287395
Frankliniella occidentalis	Fo CYP12	XP052132028
Frankliniella occidentalis	Fo CYP18	XP052132697
Frankliniella occidentalis	Fo CYP24	XP052129246
Frankliniella occidentalis	Fo CYP35	XP052127336
Frankliniella occidentalis	Fo CYP40	XP052126914
Frankliniella occidentalis	Fo CYP45	XP052126387
Frankliniella occidentalis	Fo CYP46	XP052124927
Frankliniella occidentalis	Fo CYP47	XP026273658
Frankliniella occidentalis	Fo CYP48	XP026287901
Frankliniella occidentalis	Fo CYP49	XP026284054
Frankliniella occidentalis	Fo CYP50	XP052123820
Homo sapiens	Hs CYP1	NP000766
Homo sapiens	Hs CYP2	NP000752
Homo sapiens	Hs CYP3	NP000764
Homo sapiens	Hs CYP4	NP000762
Homo sapiens	Hs CYP5	NP000761
Mus musculus	Mm CYP1	NP083255
Mus musculus	Mm CYP2	NP067257
Mus musculus	Mm CYP3	NP034137
Mus musculus	Mm CYP4	NP034123
Mus musculus	Mm CYP5	NP034138
Rattus norvegicus	Rn CYP1	NP786942
Spodoptera exigua	Se CYP32	QWM97828

Table S2. GenBank accession numbers and abbreviations used for phylogenetic analysis of sEH

**Species**	**Abbreviation**	**Accession number**
Aedes aegypti	Aaeg PA	AAEL006354
Aedes aegypti	Aaeg PB	EAT42095
Aedes aegypti	Aaeg1	XP001651935
Anopheles gambiae	Agam1	EAA10122
Anopheles gambiae	Agam2	EAL40266
Anopheles gambiae	Agam3	EAL40265
Bombyx mori	mBmori	BAH97088
Bombyx mori	Bm	XP004927786
Bombyx mori	Bmor X1	XP004927786
Bombyx mori	Bmor X2	XP021204834
Caenorhabditis elegans	Cele	CCD61875
Drosophila melanogaster	Dmel	BAD04047
Frankliniella occidentalis	Fo-EH1	XP052128701
Frankliniella occidentalis	Fo-EH2	XP026280634
Frankliniella occidentalis	Fo-EH3	XP026275638
Frankliniella occidentalis	Fo-EH4	KAE8745912
Gallus	Ggal	AAZ38461
Helicoverpa armigera	Harm	XP021183905
Homo sapiens	Hsap	AAC41694
Homo sapiens	Hsap Hs	NP775838
Mus musculus domesticus	Mmus	AAA37555
Plutella xylostella	Pxyl	XP011556182
Rattus norvegicus	sRnor	CAA46211
Rattus norvegicus	mRnor	AAA41585
Spodoptera exigua	Se-JHEH	ABD85119
Spodoptera exigua	Se-sEH	QWM97829
Spodoptera litura	Slit	XP022829648
Strongylocentrotus purpuratus	Spur	NP001121538

Table S3. Sequences of primers and prob used in this study and their PCR conditions

Primer	Use	Orientation	Sequence (5'-3')	Annealing temperature (ºC)	Amplicon (bp)
EF1	qPCR	Forward	TCA AGG AAC TGC GTC GTG GAT	52	130
		Reverse	ACA GGG GTG TAG CCG TTA GAG		
Fo-CYP24	qPCR	Forward	CGCCGCACTCAAGAACT	54	304
		Reverse	CAAGAAGGGCAGGAAGTTGA		
Fo-CYP40	qPCR	Forward	CACCCTCATCTTCCTGAACAAC	54	248
		Reverse	TGTAGTCTCGGCCTTGGATAG		
Fo-sEH1	qPCR	Forward	AAGTTCATTCTTGTTGCACACG	52	388
		Reverse	TTGAAGGCCTCGATGTCTTCC		
Fo-sEH2	qPCR	Forward	TTCTTCCTGTTCCAAGTGCCTT	52	752
		Reverse	TTCTCATCCGGAGTGATTCCTTC		
Fo-sEH3	qPCR	Forward	GGTAGAGATCGTCCCCTTATTC	52	348
		Reverse	ACAAGGAACCATCCCAGAAG		
Fo-JHEH	qPCR	Forward	AACATCCCCGACTCAGTGATC	52	523
		Reverse	TTGACGTGAATGAAGTGGAGG		
T7- Fo-CYP24	RNAi	Forward	TAATACGACTCACTATAGGGAGACGCCGCACTCAAGAACT	54	354
		Reverse	TAATACGACTCACTATAGGGAGACAAGAAGGGCAGGAAGTTGA		
T7- Fo-CYP40	RNAi	Forward	TAATACGACTCACTATAGGGAGACACCCTCATCTTCCTGAACAAC	54	250
		Reverse	TAATACGACTCACTATAGGGAGATGTAGTCTCGGCCTTGGATAG		
T7- Fo-sEH1	RNAi	Forward	TAATACGACTCACTATAGGGAGATGCATTGCGCAGAAGAATCAG	52	367
		Reverse	TAATACGACTCACTATAGGGAGAGCTTAAGAAATGAACGCATTGC		
T7- Fo-sEH2	RNAi	Forward	TAATACGACTCACTATAGGGAGATTCTTCCTGTTCCAAGTGCCTT	52	800
		Reverse	TAATACGACTCACTATAGGGAGATTCTCATCCGGAGTGATTCCTTC		
T7- Fo-sEH3	RNAi	Forward	TAATACGACTCACTATAGGGAGAGGTAGAGATCGTCCCCTTATTC	52	398
		Reverse	TAATACGACTCACTATAGGGAGAACAAGGAACCATCCCAGAAG		
T7- Fo-JHEH	RNAi	Forward	TAATACGACTCACTATAGGGAGAAACATCCCCGACTCAGTGATC	52	321
		Reverse	TAATACGACTCACTATAGGGAGATTGACGTGAATGAAGTGGAGG		
Fo-Caspase1	qPCR	Forward	CCCAGAAGATGGCACATGGT	55	176
		Reverse	ACAATTGAGGGCACCTGCTT		
Fo-Caspase2	qPCR	Forward	ACTGCCACACTCATCTGCAA	55	148
		Reverse	CCACAATGCGGGGAGATGAT		
Fo-Caspase3	qPCR	Forward	ACTGAACTATGCGAGCCCAA	55	183
		Reverse	TTGGGGAAGAACTGCGACTC		
Fo-Caspase4	qPCR	Forward	CAGTGATGGGAGATAGGCCG	55	222

		Reverse	ACCCCATGAGCAAGTATGGC		
Fo-IAP	qPCR	Forward	CTAGCGATTCCCAAGGAGTATTT	52	267
		Reverse	CACACAGTTGACACAAGAAACC		
TWSV-N	qPCR	Forward	ATGTCTAAGGTTAAGCTCACTAAGGAA	52	777
		Reverse	TTAAGCAAGTTCTGCAAGTATTGCCTG		
TWSV-NSm	qPCR	Forward	GAAGGGTCAAGGGACAATAAC	52	372
		Reverse	CCTTCCTCCTCTTCTTCAAATG		
TWSV-NSs	qPCR	Forward	GCCTGCATTCCAAACCATAAC	52	439
		Reverse	CCCTGGCAAAGTCTATCTTCC		
T7-TWSV-N	RNAi	Forward	TAATACGACTCACTATAGGGAGAATGTCTAAGGTTAAGCTCACTAAGGAA	52	827
		Reverse	TAATACGACTCACTATAGGGAGATTAAGCAAGTTCTGCAAGTATTGCCTG		
T7-TWSV-NSm	RNAi	Forward	TAATACGACTCACTATAGGGAGAGAAGGGTCAAGGGACAATAAC	52	422
		Reverse	TAATACGACTCACTATAGGGAGACCTTCCTCCTCTTCTTCAAATG		
T7-TWSV-NSs	RNAi	Forward	TAATACGACTCACTATAGGGAGAGCCTGCATTCCAAACCATAAC	52	489
		Reverse	TAATACGACTCACTATAGGGAGACCCTGGCAAAGTCTATCTTCC		

Table S4. Probe sequences used in FISH assay

**Gene**	**Probes**	**Sequence (5′ - 3′)**
TSWV-N	Antisense	FITC-TTT-TTAAGCAAGTTCTGCAAGTATTGCCTG
	Sense	FITC-AAA-CAGGCAATACTTGCAGAACTTGCTTAA

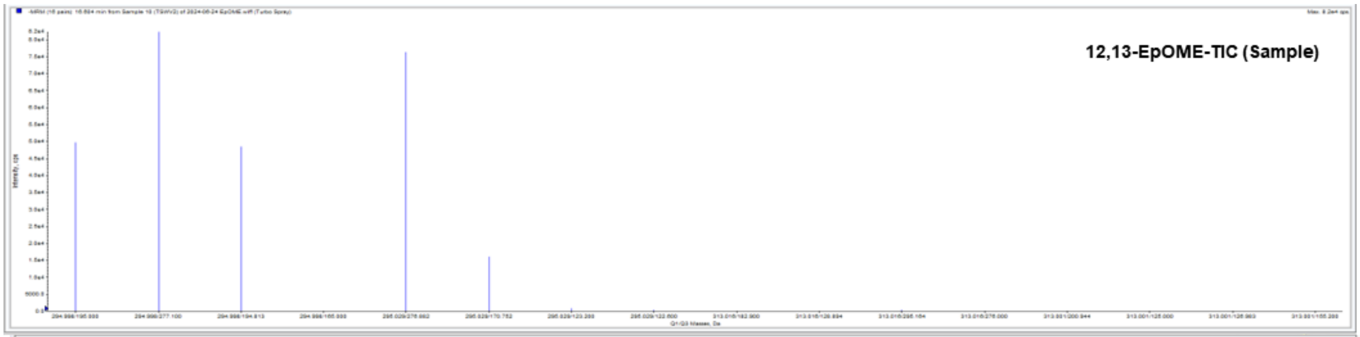
12,13-EpOME-TIC (Sample)

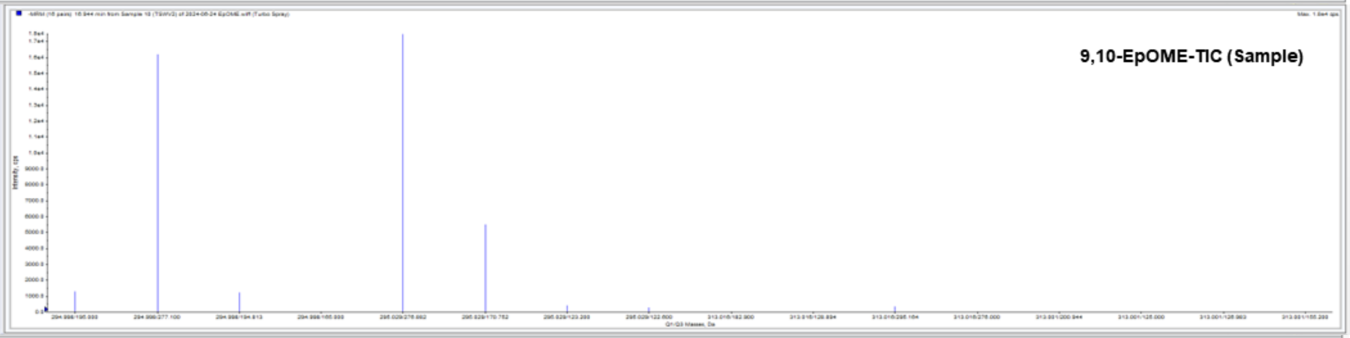
9,10-EpOME-TIC (Sample)

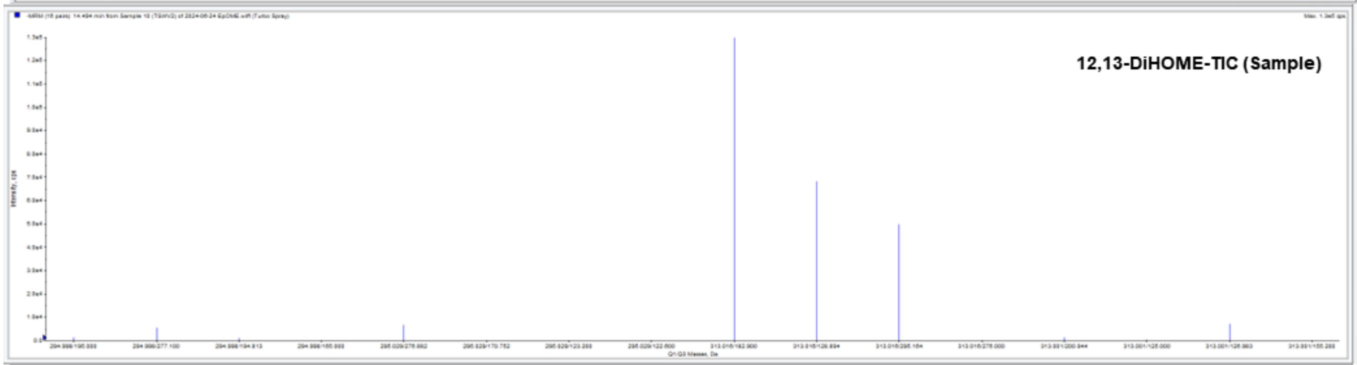
12,13-DiHOME-TIC (Sample)

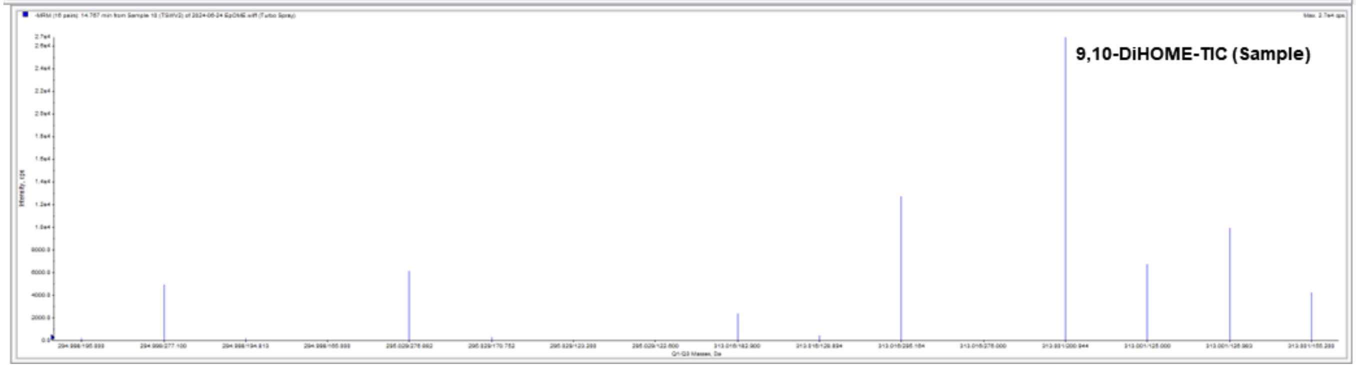
9,10-DiHOME-TIC (Sample)

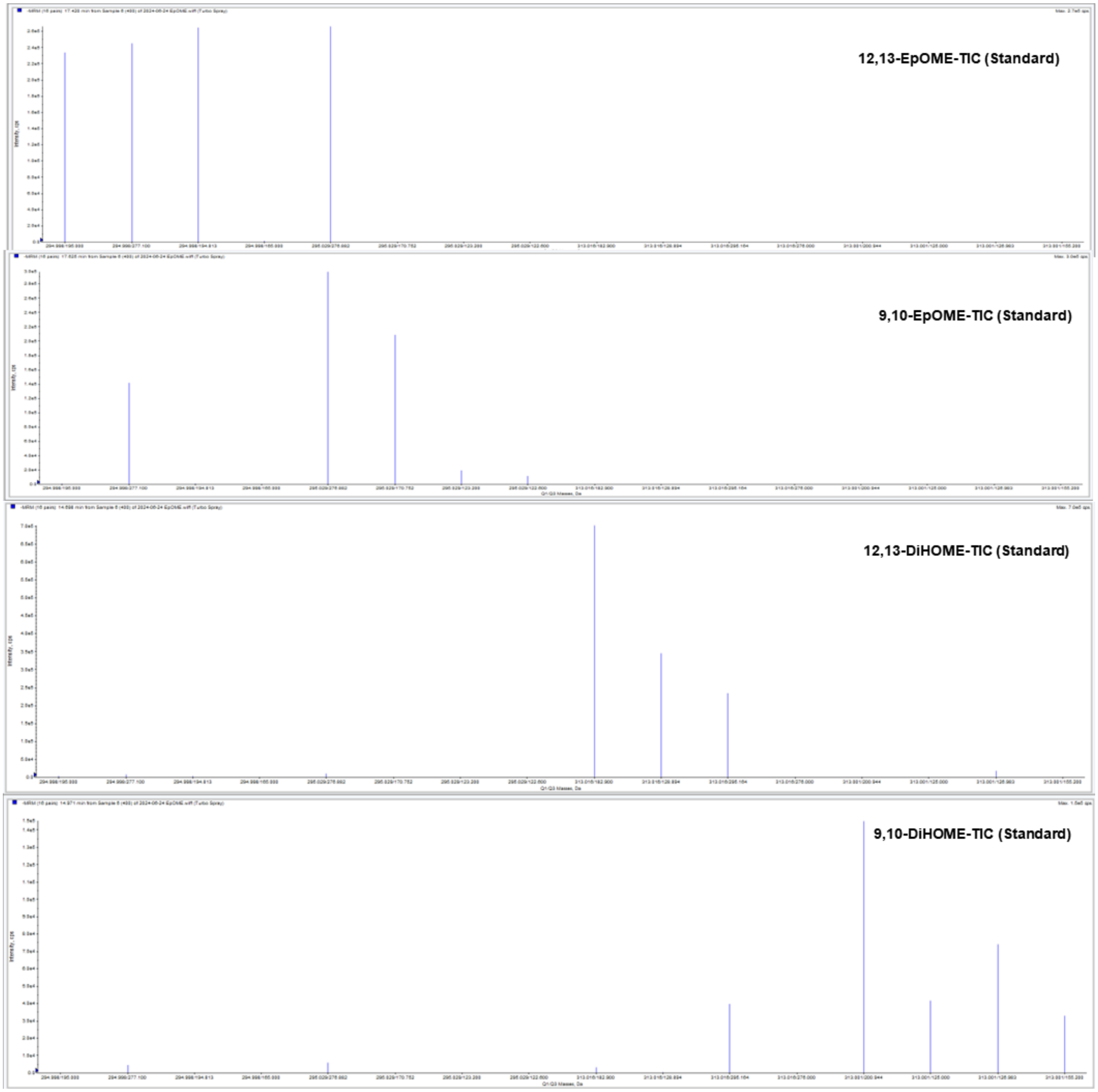
12,13-EpOME-TIC (Standard)
9,10-EpOME-TIC (Standard)
12,13-DiHOME-TIC (Standard)
9,10-DiHOME-TIC (Standard)

Figure S1

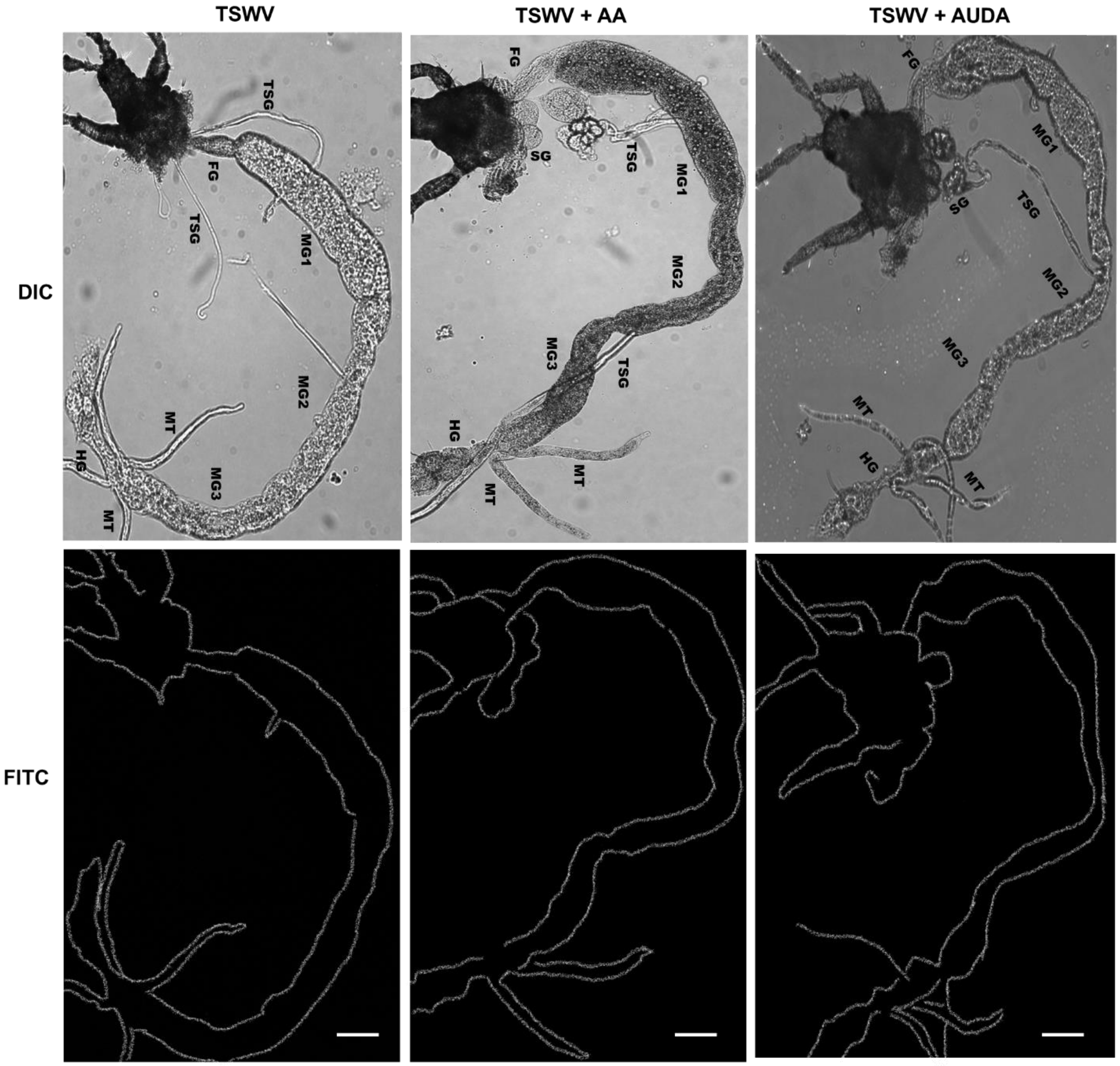

Figure S2
